# Origin, clonal diversity, and evolution of the parthenogenetic lizard *Darevskia unisexualis*

**DOI:** 10.1186/s12864-020-6759-x

**Published:** 2020-05-11

**Authors:** Andrey A. Vergun, Anastasiya E. Girnyk, Vitaly I. Korchagin, Seraphima K. Semyenova, Marine S. Arakelyan, Felix D. Danielyan, Robert W. Murphy, Alexey P. Ryskov

**Affiliations:** 1 Laboratory of Genome Organization, Institute of Gene Biology of the Russian Academy of Sciences, Vavilova Str., 34/5, Moscow, 119334 Russia; 2 Department of Biochemistry, Molecular Biology and Genetics, Moscow State Pedagogical University, M. Pirogovskaya Str., 1/1, Moscow, 119991 Russia; 3 Faculty of Biology, Yerevan State University, 1 Alex Manoogian, 0025 Yerevan, Armenia; 4 Department of Natural History, Royal Ontario Museum, 100 Queen’s Park, Toronto, ON M5S 2C6 Canada

**Keywords:** *Darevskia*, Lizards, Parthenogenesis, Clones, Clonal variation, Hybridization, Microsatellites, SNP markers, Mutations

## Abstract

**Background:**

The hybridization of female *D. raddei* and male *D. valentini* gave rise to the parthenogenetic Caucasian rock lizard *Darevskia unisexualis*. A previously identified genetic polymorphism in the species consisted of one common and two allozyme clones. Analysis of microsatellites and single nucleotide polymorphisms (SNPs) from the three species yields estimates of clonal diversity and tests the hypothesis of a single origin for *D. unisexualis*.

**Results:**

Genotyping and sequencing of four microsatellite-containing loci for 109 specimens of *D. unisexualis*, 17 *D. valentini,* and 45 *D. raddei nairensis* identified 12 presumptive clones*,* including one widespread and 11 rare clones. Most individuals in some localities had a rare clone. Clone-specific alleles in *D. unisexualis* were compared with those of the parental species. The results inferred a single hybridization event. Post-formation mutations best explain the less common clones.

**Conclusions:**

Interspecific analyses identify alleles inherited by *D. unisexualis* from its bisexual ancestors*.* SNP analyses fail to reject the hypothesis of a single interspecific origin of *D. unisexualis,* followed by microsatellite mutations in this initial clone. Microsatellites detect higher clonal diversity in *D. unisexualis* compared to allozymes and identify the likely origins of clones*.* Our approach may be applicable to other unisexual species whose origins involve interspecific hybridization.

## Background

Species of all-female, unisexual vertebrates reproduce without fertilization. Being clones, parthenospecies’ daughters are identical to their mothers, with rare exception. They are very rare in nature and usually arise via hybridization [1–5]. Some species of squamate reptiles reproduce clonally via parthenogenesis [6–8]. In some cases, the formation of parthenospecies is constrained phylogenetically [9], but in other cases not [10]. Among vertebrates, parthenogenesis was first described in the lizard genus *Darevskia* (Lacertidae) [11]. Parthenogenesis in lizards has received considerable attention, including how genetic and ecological factors play upon natural selection and speciation via hybridization, as well as the generation and evolution of genetic diversity [2, 12–16]. Hybrid parthenospecies possess the genetic diversity of their parental species [9, 10, 14] and most parthenospecies are triploids, although diploids exist, and their fixed heterozygosity results in high levels of nuclear gene diversity [17]. Sister chromatid pairing maintains heterozygosity in clones; this may offset potential reduced fitness [18, 19], but can also lead to heterozygote disadvantage and negative epistasis.

Most parthenospecies have several clones owing to mutations (especially in hypervariable microsatellite loci), multiple hybridizations from different founders, or rarely some level of genetic recombination or new hybridization events [20–22]. This variation correlates with time since hybridization, size of the area the ancestral species occupied, and ecological conditions [23–25].

Herein, we use parthenogenetic *Darevskia unisexualis* to test three hypotheses. 1) The parthenospecies has a single origin from the hybridization of paternal *D. valentini* and maternal *D. raddei* [26]. 2) Most clonal variation owes to post-hybridization mutation. And 3) analyses microsatellite loci will detect higher clonal diversity compared to allozymes. To test these hypotheses, assessments of clonal variation are essential. The extent of variation within parthenospecies can depend on the rate of clonal formation [27], ecological specialization of clonal lineages [28], historical biogeography [29], and other processes.

Parthenogenetic *D. unisexualis* was found to have three allozyme clones, based on analyses of 36 loci from three populations of *D. unisexualis* in central Armenia (*n* = 57) [26]. Rare clones occurred in two individuals and all others consisted of a common, widespread clone. Its low level of variation in mitochondrial DNA and allozymes among populations suggested that the founders of *D. unisexualis* involved very few individuals [25, 26]. However, the origin of this variation, whether owing to point mutations, insertion/deletions, multiple origins, or more complex genomic reorganization, remains unresolved, in part due to the species’ widespread distribution. The species occurs in East Anatolia and in small, isolated areas in central Armenia (Aragatsotn, Gegharkunik, Kotayk, Lori, and Shirak provinces) [30] where it prefers rocky exposures and its vertical distribution ranges from 1500 to 2300 m a.s.l. Although many populations are large, some are threatened by overgrazing and urbanization. Accordingly, this species is classified as “Near Threatened” by the IUCN and listed as “Vulnerable” in the Red Book of Armenia [30].

Paternal *D. valentini* occurs in eastern Turkey and high montane habitats (elevations of 1900–3110 m) in central Armenia and adjoining Georgia; populations are locally abundant and IUCN assessed this species as Least Concern [30]. Maternal ancestor *D. raddei* is widespread throughout central Armenia, with isolated populations in the north and in south-central portions of the country; it also occurs in adjoining Georgia and East Anatolia [31]. Like other congeners, *D. raddei* prefers stony or rocky habitats at elevations of 1000–2660 m. Individuals are usually abundant and the IUCN assessed it as “Least Concern” [30]. *Darevskia raddei* has been suggested to be a species-complex, containing the forms “*raddei*” and “*nairensis*” whose taxonomic status is still a matter of debate [25, 32, 33], and this uncertainty extends into the origins of parthenogenetic clones [34]. Notwithstanding, *D. raddei nairensis* occurs sympatrically with *D. unisexualis* at Lchap, Armenia (Gegharkunic Province) on the western margin of Lake Sevan [30]. Because the parental species of *D. unisexualis* exhibit high allozyme variation among populations [35, 36], the parthenospecies likely originated from few parental individuals [25]. Analysis of mitochondrial DNA obtained a concordant result; the four populations of *D. unisexualis* had identical sequences*,* but populations of *D. raddei* exhibit variation [25].

Our analyses of *D. unisexualis* use variation at four microsatellite-containing loci in seven Armenian populations. The same methods were used previously in our assessments of *D. dahli* [37], *D. rostombekowi* [38], and *D armeniaca* [39]. Interspecies comparisons use alleles of homologous loci from *D. unisexualis* and bisexual parents *D. valentini* and *D. raddei nairensis*. Analyses of *D. unisexualis* and its maternal parent also include partial sequences of mitochondrial cytochrome b (*CYTB*). Results show that *D. unisexualis* has a level of clonal diversity similar to ones of other parthenospecies of *Darevskia*. Analyses provide direct information about interspecific hybridization founder events, and about possible mutations in the initial hybrid clones.

## Results

All individuals of *D. unisexualis* had identical fragments of *CYTB*. The fragment assigned to haplotype of *D. raddei nairensis* from Lchashen, Armenia (data not shown; GenBank Accession No. U88613).

**Fig. 1:**
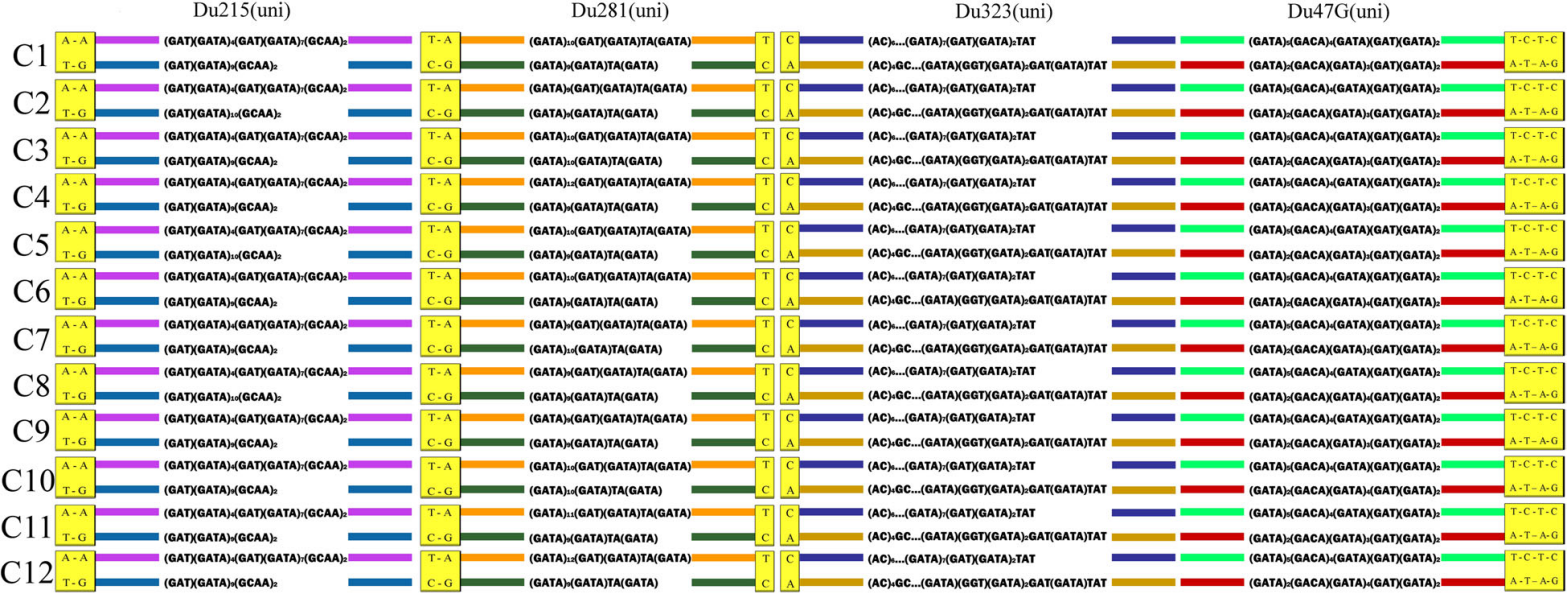
Composition of the 12 clones in 109 individuals of *Darevskia unisexualis*. Yellow squares show SNPs specific to matrilineal (*D. raddei nairensis*, above) and patrilineal (*D. valentini*, below) ancestors. Colored bars denote allele combinations of microsatellite loci Du215, Du281, Du323, and Du47G derived from maternal (above) and paternal (below) ancestors. Both alleles shown for variable loci

Each microsatellite locus in individuals of *D. unisexualis* had two alleles. Both length and structure of the alleles differed within individuals. Further, the flanking regions of the alleles had single nucleotide polymorphisms (SNPs) in fixed positions (Fig. 1 and Table S1). All clones had identical combinations of parent-specific SNPs for all loci, which was consistent with an origin from a single interspecies hybridization event; these results did not reject the first hypothesis that a single hybridization event gave rise to *D. unisexualis*. Further, the alternative hypothesis of multiple origins was rejected because *D. unisexualis* did not share alleles with multiple variants of either parental species. Assuming this to be true, then we could not reject the second hypothesis that most clonal variation owed to post-hybridization mutation.

Microsatellite analyses detected greater variation than reported for allozymes [26]. Loci Du215(uni) (*D. unisexualis*) and Du47G(uni) had three alleles, Du281(uni) had six, and Du323(uni) had two alleles (Table S1). In the paternal parent, Du215(val) (*D. valentini*) was homozygotic*,* as was Du47G(rad) (*D. raddei nairensis*) in the maternal parent. Du281(val) had five alleles, Du323(val) had six, Du47G(val) had 10, Du215(rad) had two, Du281(rad) had 11, and Du323(rad) had two. Alleles in parental *D. valentini* and *D. r. nairensis* contained microsatellite clusters and the flanking regions had SNPs at fixed positions. Six of the 14 alleles in *D. unisexualis* matched perfectly to parental alleles. Alleles Du215(uni)2,3 and Du47G(uni)1 differed from their parental species in their flanking region SNPs, and Du215(uni)2,3 differed in microsatellite repeat structure. The absence of these alleles in the parental species may have owed to sampling artifacts or genetic recombination in *D. unisexualis*.

Variation in SNPs and microsatellites resulted in 12 clones (Fig. 1 and Table 1). Clone C1 (clone 1) was found in all populations and it occurred in 37 individuals (33.9% total cohort). All other clones occurred in one or two populations only. Clone C2 occurred only at Artavaz (*n* = 28; 25.7% total cohort), C4 at Noratus only (*n* = 14; 12.8% total cohort), and C3 dominated at Lchap (n = 14; 12.8% of total cohort). Clones C5–C12 occurred in one or two populations and were found in 1–3 individuals (*n* = 16; 14.6% total cohort). Clones C10–C12 were represented by only one individual each. Because allozyme analyses only resolved three clones, our microsatellite results failed to reject the third hypothesis that microsatellites will detect more clones than allozymes.

**Table 1:** Clones, clone composition, sample size, distribution of clones among populations, and diversity of alleles in *D. unisexualis*. For clone composition, allelic notation is (allele number in *D. nairensis* + allele number in *D. valentini*). Alleles shown in Fig. 1

Clone	Clone composition	Population							Number of individuals (clone frequency)
		Artavaz	Hrazdan	Kuchak	Lchap	Noratus	Sevan	Tsovak	
C1	Du215(1 + 3) + Du281(3 + 6) + Du323(1 + 2) + Du47G(1 + 3)	1	4	4	2	1	2	23	37 (0.339)
C2	Du215(1 + 2) + Du281(5 + 6) + Du323(1 + 2) + Du47G(1 + 3)	28	0	0	0	0	0	0	28 (0.257)
C3	Du215(1 + 3) + Du281(3 + 4) + Du323(1 + 2) + Du47G(1 + 3)	0	0	4	10	0	0	0	14 (0.128)
C4	Du215(1 + 3) + Du281(1 + 6) + Du323(1 + 2) + Du47G(1 + 3)	0	0	0	0	14	0	0	14 (0.128)
C5	Du215(1 + 2) + Du281(3 + 6) + Du323(1 + 2) + Du47G(1 + 3)	3	0	0	0	0	1	0	4 (0.037)
C6	Du215(1 + 3) + Du281(3 + 6) + Du323(1 + 2) + Du47G(1 + 2)	0	0	0	1	0	0	2	3 (0.027)
C7	Du215(1 + 3) + Du281(4 + 5) + Du323(1 + 2) + Du47G(1 + 3)	0	0	2	0	0	0	0	2 (0.018)
C8	Du215(1 + 2) + Du281(5 + 6) + Du323(1 + 2) + Du47G(1 + 2)	2	0	0	0	0	0	0	2 (0.018)
C9	Du215(1 + 3) + Du281(5 + 6) + Du323(1 + 2) + Du47G(1 + 3)	0	1	1	0	0	0	0	2 (0.018)
C10	Du215(1 + 3) + Du281(3 + 4) + Du323(1 + 2) + Du47G(1 + 2)	0	0	1	0	0	0	0	1 (0.009)
C11	Du215(1 + 3) + Du281(2 + 6) + Du323(1 + 2) + Du47G(1 + 3)	0	0	0	0	1	0	0	1 (0.009)
C12	Du215(1 + 3) + Du281(1 + 6) + Du323(1 + 2) + Du47G(1 + 2)	0	0	0	0	1	0	0	1 (0.009)
Total number of individuals		34	5	12	13	17	3	25	109
Total number of clones Clone diversity (%)		4 (11.8)	2 (40.0)	5 (41.7)	3 (23.1)	4 (23.5)	2 (66.7)	2 (8.0)	12

The average values of allelic richness for individual loci varied significantly within the species. Locus Du47G had one allelic variant in all populations of *D. raddei nairensis* (Table S2), as did Du215 in *D. valentini* (Table S3). However, the polymorphic loci of the parental species often had greater allelic richness than *D. unisexualis*. Allelic richness of Du281 in populations of *D. raddei nairensis* (6.01 ± 0.24) was significantly higher (*p* < 0.05) than in *D. unisexualis* (2.42 ± 0.17), as was allelic richness of Du47G (3.36 ± 0.24) in *D .valentini* versus *D. unisexualis* (2.10 ± 0.04) (p < 0.05). Nevertheless, the average values of total allelic richness of all loci did not differ significantly among all species (*p* > 0.05) due to homozygosity of some loci of the parental species.

**Fig. 2:**
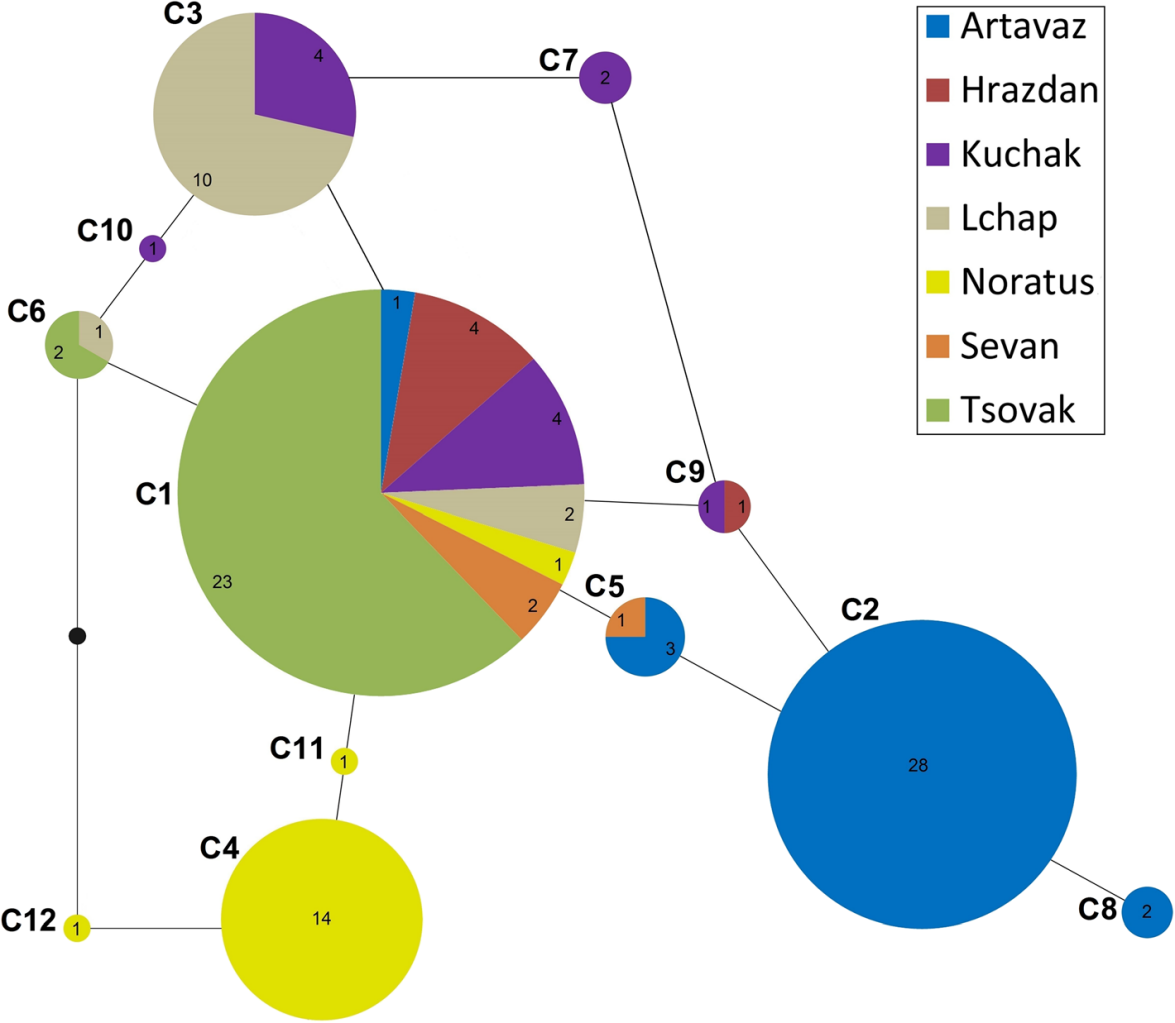
A statistical parsimony (TCS) network showing the geographic associations of the 12 clones in parthenogenetic *Darevskia unisexualis.* Analyses used differences in the number of repeats, but not indels. Number of individuals in populations given in pie slices

The TCS network placed the most common clone (C1) in a central location with respect to the other, less common clones. The other clones differed from it by one or two mutations only (Fig. 2). Within populations, clonal diversity in *D. unisexualis* ranged from 8.0 to 66.7% (Table 1). The highest levels were observed at Sevan, which had two clones in three individuals, and at Kuchak, in which the 12 individuals had one common and four rare clones. With two clones in 25 individuals, Tsovak had the lowest level of genotypic diversity. The number of alleles varied from 2 to 4 and allelic richness ranged from 1.98 to 3.00 (Table 2). Tsovak (Du281 and Du47G), Hrazdan (Du215 and Du323), and Kuchak (Du323 and Du281) had the highest values of allelic richness.

**Table 2:** Microsatellite diversity for seven populations of *D. unisexualis*

Locus	Population	Alleles (N)	R_S_
Du215	Lchap	2	2.06
	Kuchak	2	2.00
	Tsovak	2	1.99
	Noratus	2	1.99
	Artavaz	3	1.98
	Hrazdan	2	3.00
	Sevan	3	1.98
	Total	3	3.00
	Mean ± SE	2.29 ± 0.18	2.14 ± 0.14
Du281	Lchap	3	2.28
	Kuchak	4	2.60
	Tsovak	2	3.27
	Noratus	4	2.52
	Artavaz	3	2.32
	Hrazdan	3	2.00
	Sevan	2	1.98
	Total	6	6.00
	Mean ± SE	3 ± 0.31	2.42 ± 0.17
Du323	Lchap	2	1.98
	Kuchak	2	2.00
	Tsovak	2	1.99
	Noratus	2	1.99
	Artavaz	2	1.98
	Hrazdan	2	2.00
	Sevan	2	1.98
	Total	2	2.00
	Mean ± SE	2 ± 0.00	1.99 ± 0.00
Du47G	Lchap	3	2.14
	Kuchak	3	2.00
	Tsovak	3	2.23
	Noratus	3	2.21
	Artavaz	3	2.15
	Hrazdan	2	2.00
	Sevan	2	2.20
	Total	3	3.00
	Mean ± SE	2.71 ± 0.18	2.13 ± 0.04

*N* number of alleles, *R*_*S*_ allelic richness

Population genetic indices for four populations of *D. raddei nairensis* (42 individuals) were given in Table S2. Populations Ayrivank (*n* = 2) and Bjni (*n* = 1) were excluded from analyses owing to small sample sizes. Observed heterozygosity ranged from 0.40 to 1.00 (average 0.60–1.00 depending on locus), and this was similar to expected heterozygosity, which ranged from 0.32 to 0.83 (average 0.32–0.80 depending on locus). From 1 to 10 alleles were observed, depending on locus and population. Depending on locus and population, allelic richness varied from 1 to 6.67. Du281 at Pyunik had highest value of allelic richness. Expected heterozygosity was greatest in Lchashen (Du281). Population genetic indices for *D. valentini* (17 individuals) were calculated previously [39] and are presented in the Table S3.

## Discussion

Within *D. unisexualis*, the two rare allozyme clones were hypothesized to have resulted via post-formation mutation of the preexisting common clone [26]. This explanation is in accordance with Parker et al.’s model [24] that little allozyme and mtDNA variation exists in species having a single hybridization origin. Spatially, one widespread clone is common, but a few rare clones also exist. This pattern holds for other parthenogenetic Caucasian rock lizards [40, 41], as well as other parthenogenetic lizards [42]. Comparatively, parthenogenetic *D. dahli* and *D. armeniaca* also have one common clone and several rare ones [40]. Only parthenogenetic *D. rostombekowi* exhibited a single allozyme clone [43]. The level of diversity in *D. unisexualis* was also similar to that found in parthenogenetic *Aspidoscelis neomexicanus* [44], which also has a hybrid origin. In contrast, parthenogenetic *Heteronotia binoei* was reported to have much higher variation [42].

Our microsatellites and SNPs reveal higher levels of clonal diversity in parthenospecies of *Darevskia* than allozymes did. Analyses involving 109 individuals of *D. unisexualis* from seven populations in Armenia identify 12 clones that differ in their frequencies and population distribution. Analyses of 35 allozyme loci in parthenogenetic *D. dahli* [40], *D. rostombekowi* [43], and *D. armeniaca* [45] resolved five, one, and four clones, respectively, while our genomic approach resolves 11 clones in *D. dahli* [37], five in *D. rostombekowi* [38], and 13 in *D. armeniaca* [39]. Thus, assessments of microsatellites discover more variation than allozymes.

Analyses cannot reject the hypothesis of one hybridization event [26] forming *D. unisexualis* due to identical SNPs in flanking regions of the microsatellite loci and a single mitochondrial haplotype. However, it remains possible that ancestral parents experienced back crossings. Clones C1–C12 differ from each other only by microsatellite sequences. Variation in lengths of microsatellite alleles surely owes to the high rate of indels, which can occur in one generation [46]. However, future analyses of additional populations of *D. unisexualis* can test for the possibility of multiple origins, which could account for geographic patterns of alleles, as opposed to mutations within geographic regions. It is particularly important to sample populations from geographically distant locations in Turkey to differentiate between scenarios of dispersal and multiple origins.

Our analyses detect high genotypic diversity in parthenogenetic *D. unisexualis* similar to those found in parthenogenetic *D. dahli* and *D. armeniaca*. Variation in clone frequency could result from independent origins of a unisexual species because of geographic variation in the ancestors. Clones such as C2 and C4 appear to be restricted to a single population, and C3 dominates in one population. Unique clones also dominate in some populations of parthenogenetic *D. dahli* [37] and *D. armeniaca* [39]. All populations have C1 and the other clones differ from it by one or two microsatellite repeats only. Thus, the presence of these clones is more likely due to post-formation mutations [46], limited dispersal, and genetic drift. Owing to its widespread and ubiquitous distribution, C1 is likely ancestral in *D. unisexualis* (Table 1) [24]. All other clones have restricted geographic distributions.

Identification of the original area of hybridization can lead to insights and assessments of dispersal, especially when combined with dating and associated landscape models. Such analyses can lead to predictions about how climate change will affect the species. However, the exact region where C1 originated remains unknown. The highest values of clonal diversity occur in Sevan and Kuchak, and both populations are candidate sites for the origin of C1. Clones at Kuchak have origins via microsatellite mutations at Du281 and Du47G, while those at Sevan arose through mutation at Du215. Kuchak is also a contact zone of hybridization between *D. unisexualis* and *D. valentini* [47, 48]. Notwithstanding, *D. raddei nairensis* occurs sympatrically with *D. unisexualis* on the western margin of Lake Sevan [30]. This suggests two scenarios for the origin of *D. unisexualis*. First, the initial C1 arose in the Kuchak region, and then these lizards dispersed eastwardly to other regions (Artavaz, Lchap, Noratus, and other populations). Alternatively, the population at Sevan may have dispersed to western and southern areas. The site of origin remains uncertain, especially since it was proposed to have occurred on the slopes of Mount Aragats [34].

Parthenospecies of *Darevskia* appear to have evolved recently [10]. Relative to the parental species, they exhibit great mtDNA similarity and low levels of intraspecific variation. *Darevskia unisexualis* may have originated about 5000 years ago [9], or along with other parthenospecies approximately 200,000–70,000 years ago [34]. Regardless, dispersal resulted in widespread distributions involving many ecological niches.

Because it is not possible to root the network with an outgroup, statistical parsimony network (Fig. 2) has no evolutionary direction. Accordingly, we cannot be certain about the identity the primitive allele. Further, the implied reticulation results in most alleles having equal likelihoods of association with others. These are not inconsequential concerns [49]. The only seemingly unquestionable association is C8 being derived from C2; most other associations remain possible. Regardless, the most widespread allele is consistent with C1 being ancestral.

In summary, microsatellite genotyping analyses [37–39, this study] suggest that clonal diversity in parthenogenetic *D. unisexualis* and *D. rostombekowi*, which originated via a single hybridization event, owes to mutations in the initial clones. Similarly, post-formation mutations add to diversity in *D. dahli* and *D. armeniaca*, both of which originated via a few hybridizations.

## Conclusion

Analyses of four microsatellite loci and single nucleotide polymorphisms (SNPs) in their flanking regions reveal 12 presumptive clones in parthenogenetic *D. unisexualis*, including one widespread common and 11 rare clones. Assessments confirm that formation of the parthenospecies resulted from the hybridization of female *D. raddei nairensis* and male *D. valentini*. Several overall rare clones are numerous and dominate in some populations. Clonal diversity in *D. unisexualis* appears to result from microsatellite mutations in the initial clone. Parent-specific microsatellite and SNP markers identify multiple clones that allozymes could not. This approach should prove to be equally applicable to detailing the origin and variation of other unisexual species.

## Methods

**Fig. 3:**
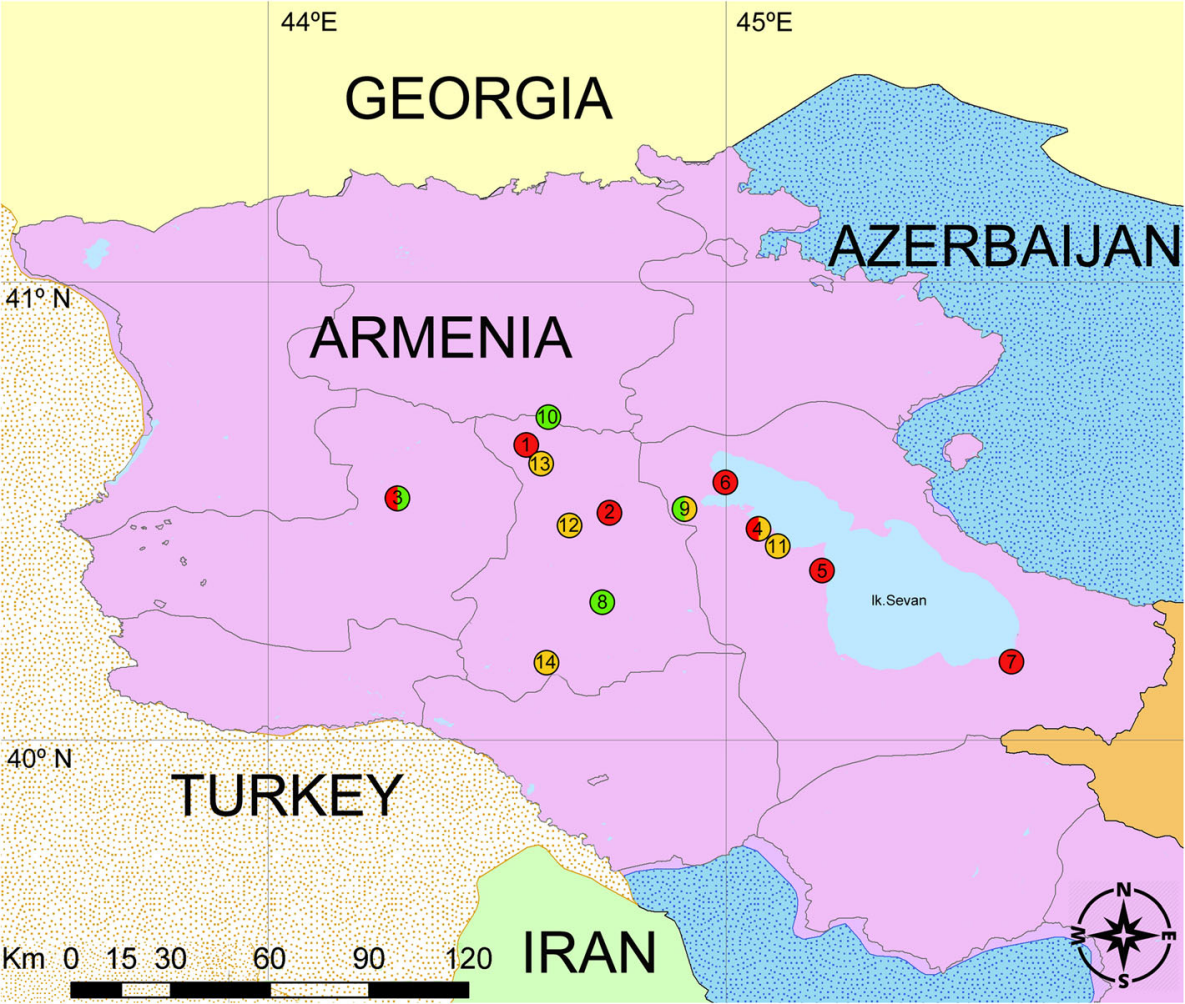
Collection localities of parthenogenetic *Darevskia unisexualis* (shown in red) and their paternal species *D. valentini* (green) and maternal *D. raddei nairensis* (yellow). Numbers indicate populations: 1 − Artavaz (Hankavan); 2 – Hrazdan; 3 – Kuchak; 4 – Lchap; 5 – Noratus; 6 – Sevan; 7 – Tsovak; 8 − Hatis (Geghama Mountains); 9 – Lchashen; 10 − Tezh (Pambak Ridge); 11 – Ayrivank; 12 − Bjni; 13 − Pyunik (Pambak Ridge); and 14 − Yerevan. A licensed version ArcGIS Desktop 10.4.1 (http://desktop.arcgis.com) was used to create the map

DNA samples were taken from seven populations of parthenogenetic *D. unisexualis* (*n* = 109). Analyses also included its parental species: six populations of matrilineal *D. raddei nairensis* (*n* = 45), and four populations of *D. valentini* (*n* = 17). All samples were from Armenia (Table 3 and Fig. 3).

**Table 3:** Species, populations, and samples of *Darevskia* used in this study

Species	(Map no.) Population	Coordinates	*N* tail tips	*N* blood	*N*_(locality)_	*N*_(species)_
*D. unisexualis*	(1) Artavaz (Hankavan)	40.622278 N 44.580944 E	14	20	34	109
	(2) Hrazdan	40.503493 N 44.748097 E	5	0	5	
	(3) Kuchak	40.530503 N 44.284286 E	2	10	12	
	(4) Lchap	40.467333 N 45.062083 E	2	11	13	
	(5) Noratus	40.377694 N 45.211667 E	0	17	17	
	(6) Sevan	40.564171 N 45.010575 E	3	0	3	
	(7) Tsovak	40.179167 N 45.622972 E	15	10	25	
*D. valentini*	(8) Hatis (Geghama Mountains)	40.304142 N 44.727975 E	0	4	4	17
	(3) Kuchak	40.530503 N 44.284286 E	0	2	2	
	(9) Lchashen	40.512756 N 44.900894 E	0	5	5	
	(10) Tezh (Pambak Ridge)	40.702244 N 44.608556 E	0	6	6	
*D. raddei nairensis*	(11) Ayrivank	40.433972 N 45.107556 E	0	2	2	45
	(12) Bjni	40.461833 N 44.652056 E	0	1	1	
	(9) Lchashen	40.512756 N 44.900894 E	0	14	14	
	(13) Pyunik (Pambak Ridge)	40.613861 N 44.585111 E	0	17	17	
	(4) Lchap	40.467333 N 45.062083 E	0	5	5	
	(14) Yerevan	40.176944 N 44.602583 E	0	6	6	
Totals						

*N* Number of individuals

We used the tips of tails of museum specimens in the herpetological collection of Yerevan State University, as well as a few blood samples collected in 2018 (Table 3). Yerevan State University approved all work with the lizards, which adhered strictly to ethical guidelines. Blood samples were obtained by removing tail tips, which autotomize, and the lizards were then released at the site of collection. DNA extraction used the standard phenol–chloroform method with proteinase K, and resuspension in TE buffer, pH 8.0.

PCR-amplification of tetranucleotide microsatellite loci Du215, Du281, Du323, and Du47G used established primers [37–39, 50]. The procedures for isolating and sequencing of individual allelic PCR-amplifications from polyacrylamide gels were carried out as described previously [37, 46].

A GenePak PCR Core Kit (Isogene) was used for amplifications in a 20 μl reaction volume, which included approximately 50 ng of DNA and 1 μM of each primer. PCR amplification conditions were used as described previously [39]. Both allelic PCR products of a locus were visualized by electrophoresis in 8% native (nondenaturating) polyacrylamide gel and then excised, purified and sequenced in both directions as previously described [39].

The number of alleles (allelic richness, *R*_*S*_) was adjusted for sample size. Expected heterozygosity was calculated by using an in-house R programming language script (available at https://github.com/andrewgull/PopGenScripts) employing packages Poppr and Mmod [51–53].

As before [54], a statistical parsimony haplotype network was calculated using TCS v.1.21 to visualize geographic distribution of clones and overall similarity. Notwithstanding, homologous alleles in parthenogenetic clones had linear arrangements via repeat number with little or no recombination. Thus, our coding (Table 4) considered gaps as a fifth state [37, 55].

**Table 4:** Coding matrix for the TCS network of alleles for *D. unisexualis*

Clone	Locus				Polymorphic Concatenated Sequence Sites					
	Du215	Du281	Du323	Du47G	1	2	3	4	5	6
1	4A/9A	10A/9A	7A/2A	5A/3A	–	A	–	–	–	–
2	4A/10A	9A/9A	7A/2A	5A/3A	A	–	–	–	–	–
3	4A/9A	10A/10A	7A/2A	5A/3A	–	A	–	–	A	–
4	4A/9A	12A/9A	7A/2A	5A/3A	–	A	A	A	–	–
5	4A/10A	10A/9A	7A/2A	5A/3A	A	A	–	–	–	–
6	4A/9A	10A/9A	7A/2A	5A/4A	–	A	–	–	–	A
7	4A/9A	9A/10A	7A/2A	5A/3A	–	–	–	–	A	–
8	4A/10A	9A/9A	7A/2A	5A/4A	A	–	–	–	–	A
9	4A/9A	9A/9A	7A/2A	5A/3A	–	–	–	–	–	–
10	4A/9A	10A/10A	7A/2A	5A/4A	–	A	–	–	A	A
11	4A/9A	11A/9A	7A/2A	5A/3A	–	A	A	–	–	–
12	4A/9A	12A/9A	7A/2A	5A/4A	–	A	A	A	–	A
GATA- Link Variability	4/9, 10	9–12/9, 10	7/2	5/3, 4						

We amplified and sequenced a 320 bp fragment of mitochondrial *CYTB* for 17 specimens of *D. unisexualis*, which amounted to 2–3 individuals from each population, as well as eight specimens of matrilineal ancestor *D. raddei nairensis* from six populations (1–2 individuals from each population). *CYTB* was chosen because it was used previously for the same species comparisons [6, 56]. PCR and sequence data were generated as using primers L14841 (5′-CCATCCAACATCTCAGCATGATGAAA-3′) and H15149 (5′-GCCCCTCAGAATGATATTTGTCCTCA-3′) [6, 57]. Amplification followed previous research [55]. An Applied Biosystems 3730 DNA Analyzer was used, and data were aligned using BioEdit v.7.0 [58].

## Supplementary information

**Additional file 1: Table S1.** Allelic variation of microsatellite containing loci in *Darevskia unisexualis*, *D. valentini*, and *D. raddei nairensis*.

**Additional file 2: Table S2.** Population indices of gene diversity for four loci in four sampled populations of *D. raddei nairensis*.

**Additional file 3: Table S3.** Population indices of gene diversity for four loci in four sampled populations of *D. valentini*.

## Data Availability

All data generated or analyzed during this study are included in this published article and its supplementary information files. All unique de novo sequences were deposited in GenBank (KX258628–KX258641; GU972551; KM573739; KM573746; KM573717–KM573727; KM573749–KM573751; KM573753; KM573755–KM573759; KM573761–M573762; HM014002–HM014003; MH187990–MH187999; MN072617). Comparative data downloaded from GenBank for *D. valentini* had accession numbers GU972551, KM573717–KM573727, and MH187990–MH187999. The *D. raddei nairensis* cytochrome b gene dataset was obtained from NCBI Genbank, deposited under the accession number U88613.

## Abbreviations

bp: Base pair
C (with number): Clone (number)
*CYTB*: Mitochondrial gene encoding cytochrome b
DNA: Deoxyribonucleic acid
H_E_: Expected heterozygosity
H_O_: Observed heterozygosity
IUCN: International Union for Conservation of Nature’s Red List
mtDNA: Mitochondrial DNA
N: Number of individuals or of alleles
P: Probability
PCR: Polymerase chain reaction
*R*_*S*_: Allelic richness
SE: Standard error
SNP: Single nucleotide polymorphisms
TCS: Tata Consultancy Services
TE buffer: Tris and EDTA buffer
