# Supplementary material for: Origin, clonal diversity, and evolution of the parthenogenetic lizard Darevskia unisexualis
Source: BMC Genomics. 2020 May 11;21:351. doi: 10.1186/s12864-020-6759-x (PMC7216553; doi:10.1186/s12864-020-6759-x)
Supplement: Supplementary file 1 — Additional file 1: Table S1. Allelic variation of microsatellite containing loci in Darevskia unisexualis, D. valentini, and D. raddei nairensis. [file 12864_2020_6759_MOESM1_ESM.pdf]

Table S1 Allelic variation of microsatellite containing loci in *Darevskia unisexualis*, *D. valentini*, and *D. raddei nairensis*

Allelic variant	Size (bp)	Structure of microsatellite cluster	SNP (nucleotide position, N)
**Du215(uni)1 (maternal)**	227	5’ **GAT(GATA)$_4$GAT(GATA)$_7$(GCAA)$_2$ 3’**	**A (-69), A (-42)**
**Du215(uni)2 (paternal)**	220	5’ **GAT(GATA)$_{10}$(GCAA)$_2$ 3’**	**T (-69), G (-42)**
**Du215(uni)3 (paternal)**	216	5’ **GAT(GATA)$_9$(GCAA)$_2$ 3’**	**T (-69), G (-42)**
Du215(val)1	192	5’ GAT(GATA)$_5$ 3’	A (-69), A (-42)
Du215(nair)1	227	5’ GAT(GATA)$_4$GAT(GATA)$_7$(GCAA)$_2$ 3’	A (-69), A (-42)
Du215(nair)2	215	5’ GAT(GATA)$_4$GAT(GATA)$_4$(GCAA)$_2$ 3’	A (-69), A (-42)
**Du281(uni)1 (maternal)**	212	5’ **(GATA)$_{12}$GAT(GATA)TA(GATA) 3’**	**T (-84), A(-19), T (+15)**
**Du281(uni)2 (maternal)**	208	5’ **(GATA)$_{11}$GAT(GATA)TA(GATA) 3’**	**T (-84), A (-19), T (+15)**
**Du281(uni)3 (maternal)**	204	5’ **(GATA)$_{10}$GAT(GATA)TA(GATA) 3’**	**T (-84), A (-19), T (+15)**
**Du281(uni)4 (paternal)**	201	5’ **(GATA)$_{11}$TA(GATA) 3’**	**C (-84), G (-19) C (+15)**
**Du281(uni)5 (maternal)**	200	5’ **(GATA)$_9$GAT(GATA)TA(GATA) 3’**	**T (-84), A (-19), T (+15)**
**Du281(uni)6 (paternal)**	197	5’ **(GATA)$_{10}$TA(GATA) 3’**	**C (-84), G (-19) C (+15)**
Du281(val)1	199	5’ (GATA)$_{12}$ 3’	C (-84), G (-19) C (+15)
Du281(val)2	195	5’ (GATA)$_{11}$ 3’	C (-84), G (-19) C (+15)
Du281(val)3	191	5’ (GATA)$_{10}$ 3’	C (-84), G (-19) C (+15)
Du281(val)4	187	5’ (GATA)$_9$ 3’	C (-84), G (-19) C (+15)
Du281(val)5	183	5’ (GATA)$_8$ 3’	C (-84), G (-19) C (+15)

Du281(nair)1	214	5’ $(GATA)_2$GAGAT$(GATA)_4(GACA)_3(GATA)_4$TA(GATA) 3’	T (-84), A (-19), T (+15)
Du281(nair)2	208	5’ $(GATA)_{11}$GAT(GATA)TA(GATA) 3’	T (-84), A (-19), T (+15)
Du281(nair)3	208	5’ $(GATA)_{10}$GAT(GATA)TA$(GATA)_2$ 3’	T (-84), A (-19), T (+15)
Du281(nair)4	204	5’ $(GATA)_{10}$GAT(GATA)TA(GATA) 3’	T (-84), A (-19), T (+15)
Du281(nair)5	197	5’ $(GATA)_{10}$TA(GATA) 3’	T (-84), A (-19), T (+15)
Du281(nair)6	196	5’ $(GATA)_8$GAT(GATA)TA(GATA) 3’	T (-84), A (-19), T (+15)
Du281(nair)7	196	5’ $(GATA)_4$GAAA$(GATA)_3$GAT(GATA)TA(GATA) 3’	T (-84), A (-19), T (+15)
Du281(nair)8	195	5’ $(GATA)_{11}$ 3’	T (-84), A (-19), T (+15)
Du281(nair)9	192	5’ $(GATA)_7$GAT(GATA)TA(GATA) 3’	T (-84), A (-19), T (+15)
Du281(nair)10	188	5’ $(GATA)_6$GAT(GATA)TA(GATA) 3’	T (-84), A (-19), T (+15)
Du281(nair)11	183	5’ $(GATA)_8$ 3’	T (-84), A (-19), T (+15)
**Du323(uni)1 (paternal)**	199	5’ **$(AC)_6$...$(GATA)_7$GAT$(GATA)_2$TAT 3’**	**C (-16)**
**Du323(uni)2 (maternal)**	180	5’ **$(AC)_4$GC...(GATA)GGT$(GATA)_2$GAT(GATA)TAT 3’**	**A (-16)**
Du323(val)1	220	5’ $(AC)_6$…$(GATA)_{15}$GAT 3’	C (-16)
Du323(val)2	216	5’ $(AC)_6$…$(GATA)_{14}$GAT 3’	C (-16)
Du323(val)3	211	5’ $(AC)_6$…$(GATA)_{10}$GAT$(GATA)_2$TAT 3’	C (-16)
Du323(val)4	211	5’ $(AC)_6$…$(GATA)_5$GAT$(GATA)_2$ 3’	C (-16)
Du323(val)5	191	5’ $(AC)_6$…$(GATA)_5$(GACA)$(GATA)_4$GAT$(GATA)_2$ 3’	C (-16)
Du323(val)6	187	5’ $(AC)_6$…$(GATA)_4$GAT$(GATA)_2$ 3’	C (-16)
Du323(nair)1	184	5’ $(AC)_4$GC...(GATA)GGT$(GATA)_3$GAT(GATA)TAT 3’	A (-16)

Du323(nair)2	180	5’ $(AC)_4$GC...(GATA)GGT$(GATA)_2$GAT(GATA)TAT 3’	A (-16)
**Du47G(uni)1 (paternal)**	164	5’ **$(GATA)_5(GACA)_4$(GATA)GAT$(GATA)_2$ 3’**	**T (+7), C (+15), T (+21), C (+52)**
**Du47G(uni)2 (maternal)**	152	5’ **$(GATA)_2$(GACA)$(GATA)_4$GAT$(GATA)_2$ 3’**	**A (+7), T (+15), A(+21), G (+52)**
**Du47G(uni)3 (maternal)**	148	5’ **$(GATA)_2$(GACA)$(GATA)_3$GAT$(GATA)_2$ 3’**	**A (+7), T (+15), A(+21), G (+52)**
Du47G(val)1	211	5’$(GATA)_5$GAT$(GATA)_{14}$(GACA)(GATA)GAT$(GATA)_2$ 3’	T (+7), T (+15), T (+21), C (+52)
Du47G(val)2	200	5’ $(GATA)_{17}$(GACA)(GATA)GAT$(GATA)_2$ 3’	T (+7), T (+15), T (+21), C (+52)
Du47G(val)3	196	5’ $(GATA)_{16}$(GACA)(GATA)GAT$(GATA)_2$ 3’	T (+7), T (+15), T (+21), C (+52)
Du47G(val)4	192	5’ $(GATA)_{15}$(GACA)(GATA)GAT$(GATA)_2$ 3’	T (+7), T (+15), T (+21), C (+52)
Du47G(val)5	188	5’ $(GATA)_{14}$(GACA)(GATA)GAT$(GATA)_2$ 3’	T (+7), T (+15), T (+21), C (+52)
Du47G(val)6	184	5’ $(GATA)_{13}$(GACA)(GATA)GAT$(GATA)_2$ 3’	T (+7), T (+15), T (+21), C (+52)
Du47G(val)7	180	5’ $(GATA)_{12}$(GACA)(GATA)GAT$(GATA)_2$ 3’	T (+7), T (+15), T (+21), C (+52)
Du47G(val)8	176	5’ $(GATA)_{11}$(GACA)(GATA)GAT$(GATA)_2$ 3’	T (+7), T (+15), T (+21), C (+52)
Du47G(val)9	168	5’ $(GATA)_9$(GACA)(GATA)GAT$(GATA)_2$ 3’	T (+7), T (+15), T (+21), C (+52)
Du47G(val)10	164	5’ $(GATA)_8$(GACA)(GATA)GAT$(GATA)_2$ 3’	T (+7), T (+15), T (+21), C (+52)
Du47G(nair)1	148	5’ $(GATA)_2$(GACA)$(GATA)_3$GAT$(GATA)_2$ 3’	A (+7), T (+15), A(+21), G (+52)
