# Supplementary material for: Origin, clonal diversity, and evolution of the parthenogenetic lizard Darevskia unisexualis
Source: BMC Genomics. 2020 May 11;21:351. doi: 10.1186/s12864-020-6759-x (PMC7216553; doi:10.1186/s12864-020-6759-x)
Supplement: Supplementary file 2 — Additional file 2: Table S2. Population indices of gene diversity for four loci in four sampled populations of D. raddei nairensis. [file 12864_2020_6759_MOESM2_ESM.pdf]

Table S2 Population indices of gene diversity for four loci in four sampled populations of *D. raddei nairensis*

Locus	Population	Allele (N)	R_S	H_E	H_O
Du215	Pyunik	1	1.00	-	0.00
	Lchap	2	2.00	0.32	0.40
	Lchashen	2	2.00	0.50	1.00
	Yerevan	2	2.00	0.50	1.00
	Total	2	2.00	0.39	0.52
	Mean± SE	1.75±0.25	1.75±0.25	0.32±0.12	0.60±0.24
Du281	Pyunik	7	6.67	0.81	1.00
	Lchap	6	5.90	0.76	0.80
	Lchashen	10	6.00	0.83	0.79
	Yerevan	6	5.48	0.78	0.83
	Total	11	14	0.86	0.88
	Mean±SE	7.25±0.95	6.01±0.24	0.80±0.02	0.85±0.05
Du323	Pyunik	2	2.00	0.50	1.00
	Lchap	2	2.00	0.50	1.00
	Lchashen	2	2.00	0.50	1.00
	Yerevan	2	2.00	0.50	1.00
	Total	2	2.00	0.50	1.00
	Mean±SE	2±0.00	2.00±0.00	0.50±0.00	1.00±0.00
Du47G	Pyunik	1	1.00	-	0.00
	Lchap	1	1.00	-	0.00
	Lchashen	1	1.00	-	0.00
	Yerevan	1	1.00	-	0.00
	Total	1	1.00	-	0.00
	Mean±SE	1.00±0.00	1.00±0.00	-	0.00±0.00

N number of alleles, R_S allelic richness, H_E expected heterozygosity, H_O observed heterozygosity.
