# Supplementary material for: Origin, clonal diversity, and evolution of the parthenogenetic lizard Darevskia unisexualis
Source: BMC Genomics. 2020 May 11;21:351. doi: 10.1186/s12864-020-6759-x (PMC7216553; doi:10.1186/s12864-020-6759-x)
Supplement: Supplementary file 3 — Additional file 3: Table S3. Population indices of gene diversity for four loci in four sampled populations of D. valentini. [file 12864_2020_6759_MOESM3_ESM.pdf]

Table S3 Population indices of gene diversity for four loci in four sampled populations of *D. valentini*

Locus	Population	Alelle (N)	R_S	H_E	H_O
Du215	Lchashen	1	1.00	-	0.00
	Kuchak	1	1.00	-	0.00
	Tezh	1	1.00	-	0.00
	Adis	1	1.00	-	0.00
	Total	1	1.00	-	0.00
	Mean±SE	1±0	1.00±0.00	-	0±0.00
Du281	Lchashen	3	1.79	0.60	0.80
	Kuchak	2	2.00	0.50	0.50
	Tezh	4	2.23	0.56	0.33
	Adis	2	2.24	0.43	0.50
	Total	5	5.00	0.56	0.53
	Mean±SE	2.75±0.48	2.06±0.11	0.52±0.04	0.53±0.10
Du323	Lchashen	3	3.00	0.64	1.00
	Kuchak	3	3.00	0.83	0.50
	Tezh	2	2.3	0.55	1.00
	Adis	4	1.94	0.82	0.75
	Total	4	4.00	0.66	0.88
	Mean±SE	3±0.41	2.56±0.26	0.71±0.07	0.81±0.12
Du47G	Lchashen	5	3.43	0.82	0.80
	Kuchak	3	4.00	0.83	1.00
	Tezh	6	2.96	0.82	1.00
	Adis	6	3.05	0.89	1.00
	Total	10	12.00	0.84	0.94

Mean±SE	5±0.71	3.36±0.24	0.84±0.02	0.95±0.05

N number of alleles, R_S allelic richness, H_E expected heterozygosity, H_O observed heterozygosity
